# Type-2 Neutrosophic Markov Chain Model for Subject-Independent Sign Language Recognition: A New Uncertainty–Aware Soft Sensor Paradigm

**DOI:** 10.3390/s24237828

**Published:** 2024-12-07

**Authors:** Muslem Al-Saidi, Áron Ballagi, Oday Ali Hassen, Saad M. Saad

**Affiliations:** 1 Doctoral School of Multidisciplinary Engineering Sciences, Széchenyi István University, Egyetem tér 1, 9026 Gyor, Hungary; 2 Department of Automation, Széchenyi István University, Egyetem tér 1, 9026 Gyor, Hungary; ballagi@ga.sze.hu; 3 Ministry of Education, Wasit Education Directorate, Kut 52001, Iraq; odayali@uowasit.edu.iq; 4 Department of Information Technology, Institute of Graduate Studies and Research, Alexandria University, Alexandria 21526, Egypt; saad.darwish.pt@pua.edu.eg; 5 Department of Artificial Intelligence, Faculty of Computer Sciences and Artificial Intelligence, Pharos University in Alexandria, Canal El Mahmoudia Street, Beside Green Plaza, Alexandria 21648, Egypt

**Keywords:** uncertainty-aware soft sensor, sign language recognition, Type-2 Neutrosophic reasoning, hidden Markov model

## Abstract

Uncertainty-aware soft sensors in sign language recognition (SLR) integrate methods to quantify and manage the uncertainty in their predictions. This is particularly crucial in SLR due to the variability in sign language gestures and differences in individual signing styles. Managing uncertainty allows the system to handle variations in signing styles, lighting conditions, and occlusions more effectively. While current techniques for handling uncertainty in SLR systems offer significant benefits in terms of improved accuracy and robustness, they also come with notable disadvantages. High computational complexity, data dependency, scalability issues, sensor and environmental limitations, and real-time constraints all pose significant hurdles. The aim of the work is to develop and evaluate a Type-2 Neutrosophic Hidden Markov Model (HMM) for SLR that leverages the advanced uncertainty handling capabilities of Type-2 neutrosophic sets. In the suggested soft sensor model, the Foot of Uncertainty (FOU) allows Type-2 Neutrosophic HMMs to represent uncertainty as intervals, capturing the range of possible values for truth, falsity, and indeterminacy. This is especially useful in SLR, where gestures can be ambiguous or imprecise. This enhances the model’s ability to manage complex uncertainties in sign language gestures and mitigate issues related to model drift. The FOU provides a measure of confidence for each recognition result by indicating the range of uncertainty. By effectively addressing uncertainty and enhancing subject independence, the model can be integrated into real-life applications, improving interactions, learning, and accessibility for the hearing-impaired. Examples such as assistive devices, educational tools, and customer service automation highlight its transformative potential. The experimental evaluation demonstrates the superiority of the Type-2 Neutrosophic HMM over the Type-1 Neutrosophic HMM in terms of accuracy for SLR. Specifically, the Type-2 Neutrosophic HMM consistently outperforms its Type-1 counterpart across various test scenarios, achieving an average accuracy improvement of 10%.

## 1. Introduction

Soft sensors, also known as virtual sensors or inferential sensors, are algorithms or models that estimate process variables that are difficult, expensive, or impossible to measure directly. They utilize easily measurable variables and historical data to infer the desired measurements, providing critical insights for process control and optimization [1]. Uncertainty-aware soft sensors incorporate methods to quantify and manage the uncertainty in their predictions. This is essential because all models inherently have some level of uncertainty due to factors such as model approximation, measurement noise, and changes in process conditions. By being aware of these uncertainties, soft sensors can provide more reliable and robust predictions, enhancing decision-making processes in various industrial applications. The quality of input data from sensors or cameras can vary due to factors like lighting, background noise, and occlusions, affecting the accuracy and uncertainty estimation [2,3].

**Figure 1:**
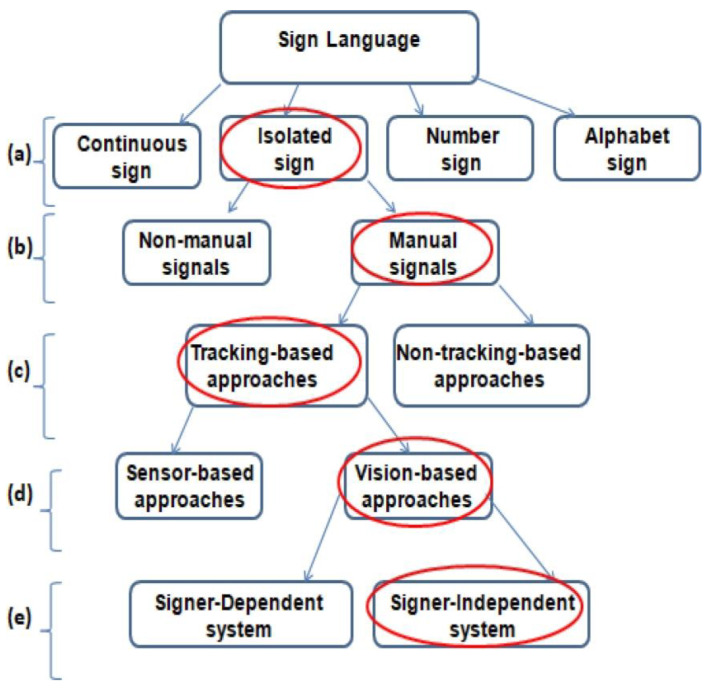
Study context: (**a**) sign categories, (**b**) sign characteristics, (**c**) sign detection techniques, (**d**) sign data acquisition approaches, and (**e**) sign classification conditions [4]. The red circles highlight the key focus areas of our study within the hierarchy of sign language recognition.

Subject-independent sign language recognition (SISLR) involves developing a system capable of recognizing sign language gestures from different individuals, without requiring the model to be specifically trained on each person’s unique signing style. This contrasts with subject-dependent systems, which typically perform better because they are trained on data from the same individuals who will be using the system (see Figure 1) [4,5]. Uncertainty in SISLR is a significant challenge, as the system needs to accurately recognize gestures from a wide range of users with varying signing styles, physical characteristics, and environmental conditions. In SISLR, model uncertainty (epistemic uncertainty) occurs when the model encounters signing styles or features it has not seen before, whereas data uncertainty (Aleatoric uncertainty) comes from the inherent noise or ambiguity in the input data. In SISLR, this could be due to poor video quality, lighting variations, occlusions, or inconsistent signing by the user. By addressing model and data uncertainty through advanced modeling techniques, robust preprocessing, and adaptive learning, SISLR systems can become more reliable and effective in real-world applications [6].

The high uncertainty associated with feature descriptions in SISLR can significantly increase the risk of misclassification. This uncertainty arises from the variability in signing styles, hand shapes, movements, and other non-manual cues across different individuals. Traditional methods, such as fuzzy logic, may struggle to accurately differentiate between types of signs under these conditions. Neutrosophic logic (NL) offers an enhanced approach by explicitly handling truth, indeterminacy, and falsity, making it particularly effective in managing the complexities of SISLR. By treating indeterminacy as a distinct dimension, NL helps address the challenges of ambiguity and contradictory information, improving the system’s ability to manage uncertain sign language inputs where the degree of truthfulness, indeterminacy, and falsity can vary widely [7,8].

**Figure 2:**
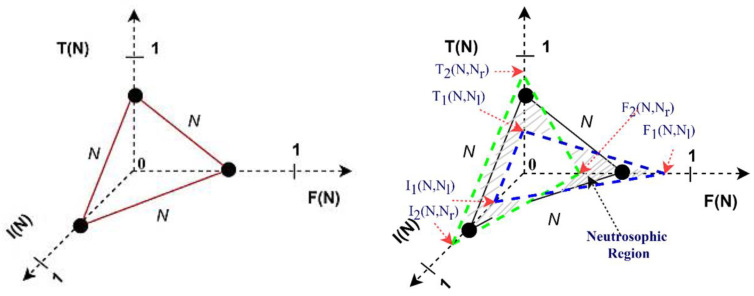
(**Left**) Type-1 neutrosophic truth T(N), indeterminacy I(N), and falsity membership functions F(N). (**Right**) Type-2 neutrosophic set membership functions. The blurred region in T2NS provides two extra memberships for truth, indeterminacy, and falsify membership functions TM, IM, and FM. The two extra memberships are $N_{l}$ and $N_{r}$; l and r are left and right shifts [7].

Type-2 Neutrosophic Logic (T2NL) with the Footprint of Uncertainty (FOU) provides many advantages when uncertainty is frequent and has to be accurately modeled. FOU inclusion makes it possible to show uncertainty more fully by including not just the degree of membership but also the associated uncertainty [7]. Compared to Type-1 Neutrosophic Logic (T1NL), T2NL offers a stronger foundation for handling imprecision and uncertainty as T2NL simulates uncertainty at the level of the membership function as much as the membership values. This makes T2NL more sophisticated in its depiction of complex uncertain data by enabling it to record uncertainty about uncertainty. Higher degrees of uncertainty in the membership functions provide better representations of situations with ambiguous boundaries between several categories [8,9,10,11]. By representing truth, indeterminacy, and falsity as intervals, T2NL can more accurately capture the variability and vagueness inherent in uncertain information (see Figure 2). This interval representation acknowledges that the exact degree of membership is often not precisely known. The range of values (the Foot of Uncertainty) provides a buffer zone that accommodates fluctuations and uncertainties in the data, leading to more robust decision-making processes. The FOU helps to absorb and mitigate the impact of noise and variability in the input data, leading to more stable and reliable outputs. By analyzing the width of the intervals, it is possible to gain insights into the degree of uncertainty present in the system, facilitating more informed and precise analyses [10,11,12].

Hidden Markov Models (HMMs) have long been a staple in sequential data analysis, including SISLR. While deep learning approaches have gained popularity due to their powerful pattern recognition capabilities, HMMs still offer several important advantages, particularly in certain contexts within SISLR [13,14,15,16]. HMMs are less computationally intensive compared to deep learning models, which require significant resources for training and inference. For real-time SISLR applications or when deploying on devices with limited computational power (e.g., mobile devices), HMMs can be more practical. In addition, HMMs can achieve reasonable performance with smaller datasets, whereas deep learning models often require large amounts of data to avoid overfitting and to generalize well. In situations where only a limited amount of sign language data is available, HMMs can be more effective, providing a good balance between performance and data requirements. In addition, HMMs, due to their simpler structure and fewer parameters, are less prone to overfitting, especially in cases where the training data are limited or noisy. However, HMMs rely on fixed probability distributions (e.g., Gaussian) to model the likelihood of observations given a hidden state. These distributions are often simplistic and may not adequately capture the complex variability and uncertainty in real-world SLR data [17]. Currently, Type-2 Neutrosophic Logic offers enhanced capabilities for handling high levels of uncertainty that can address some of the problems faced by HMMs when using Type-1 Neutrosophic Logic [18].

### 1.1. Problem Statement and Motivation

SLR systems face significant challenges in accurately interpreting signs due to the inherent variability in gesture execution, signer-specific differences, and environmental factors such as lighting and background noise. These variations introduce considerable uncertainty, making it difficult for traditional SLR models to generalize effectively, particularly in subject-independent scenarios. Additionally, the current systems often lack a mechanism to explicitly quantify and manage this uncertainty, leading to reduced performance in real-world applications. The introduction of an Uncertainty-Aware Soft Sensor Paradigm for SLR, incorporating Type-2 Neutrosophic Logic, provides a promising solution by explicitly modeling the uncertainty present in gesture recognition. By accounting for different levels of truth, indeterminacy, and falsehood, this new soft sensor approach aims to improve the system’s ability to deal with ambiguity, thereby enhancing the accuracy, reliability, and generalizability of SLR systems. This paradigm would be particularly valuable in assistive technologies, human–computer interaction, and accessibility tools for the deaf community.

### 1.2. Contribution

This paper introduces a new Uncertainty-Aware Soft Sensor Paradigm for SISLR based on a Type-2 Neutrosophic HMM, which employs the Footprint of Uncertainty (FOU) to model and manage the uncertainties inherent in gesture recognition. Unlike the conventional Type-1 Neutrosophic HMM, this approach captures deeper levels of uncertainty by considering not only truth but also indeterminacy and falsity within sign data. The use of the FOU allows the model to define dynamic boundaries for decision-making, enhancing its ability to deal with imprecise, ambiguous, and noisy input. This paradigm significantly improves subject-independent recognition, offering higher robustness and adaptability in real-world scenarios. It contributes to the field by providing a more accurate, scalable, and uncertainty-aware framework for SLR, addressing key challenges like diverse signing styles, environmental variations, and data inconsistency, making it highly applicable to assistive technologies.

The remainder of this paper consists of the following sections: Section 2 provides a literature review of relevant publications for the uncertainty-aware SISLR framework. The suggested Type-2 Neutrosophic HMM-based SISLR approach is presented in Section 3. The assessment of the suggested technique, including the results and discussion, is presented in Section 4. In Section 5, the study is concluded, and possible future directions are discussed.

## 2. Related Work

While current methods for uncertainty-aware SLR offer various ways to handle uncertainty, each comes with trade-offs [19,20,21,22,23,24]. Methods like Bayesian Neural Networks and Gaussian Processes provide excellent uncertainty quantification but at the cost of computational complexity and scalability. On the other hand, Type-1 Neutrosophic Logic and ensemble methods offer flexibility and robustness in noisy environments but may struggle with real-time processing. Emerging techniques like deep evidential regression are promising for real-time uncertainty estimation but need further exploration and optimization for widespread use in SLR.

Gaussian Processes (GPs) provide a probabilistic framework that quantifies uncertainty in SLR, making them effective for detecting ambiguous or novel gestures. Their non-parametric nature allows them to adapt to various sign data distributions without assuming a specific form. Additionally, GPs offer inherent uncertainty estimates, enhancing the model’s reliability for applications such as assistive technologies. However, GPs face scalability challenges with large datasets due to the quadratic growth of the covariance matrix, and they can be computationally intensive during training and inference. They also may struggle with high-dimensional data or complex sequences often encountered in SLR tasks [21].

Bayesian Neural Networks (BNNs) offer robust handling of uncertainty by quantifying both model (epistemic) and data (Aleatoric) uncertainties, making them adept at managing variations in signing styles, lighting conditions, and ambiguous gestures. They provide confidence intervals for predictions, which helps to avoid overconfidence and supports better decision-making in uncertain scenarios. Additionally, BNNs can reduce overfitting in smaller or imbalanced datasets. However, they come with high computational costs due to complex inference techniques like Monte Carlo sampling or variational inference, and their scalability can be problematic for large datasets. Furthermore, their emphasis on uncertainty estimation may sometimes result in lower raw accuracy compared to deterministic neural networks [23,24].

Ensemble methods, such as Bootstrap Aggregating, enhance uncertainty estimation by aggregating outputs from multiple models, which makes them effective at handling ambiguous sign inputs. They also help reduce overfitting, which is crucial for small or imbalanced datasets often seen in SLR. However, they come with increased resource demands, as training multiple models or performing multiple passes through a single model raises computational costs and can impact real-time performance. Moreover, tuning ensemble methods requires careful experimentation, and the performance gains may be marginal compared to well-optimized single models, raising concerns about the balance between performance improvements and resource use [23].

Deep evidential regression offers efficient uncertainty quantification in a single pass, making it suitable for real-time SLR without needing multiple forward passes or ensemble methods. It directly predicts uncertainty associated with each sign gesture, which improves reliability in noisy or ambiguous situations. However, the approach involves complex interpretation and tuning, requiring expertise in evidential theory and deep learning. Its adoption is still limited, and there is sparse information on its performance with large-scale or diverse datasets. Moreover, if not properly regularized, deep evidential regression models may be prone to overfitting, particularly in imbalanced or small datasets common in SLR [24,25].

Type-1 Neutrosophic Logic Models excel at handling uncertainty by representing truth, indeterminacy, and falsity, which helps to manage ambiguous or noisy sign inputs more effectively than traditional methods. They enhance decision-making by providing a framework to address unclear gestures and can be applied to various scenarios, including occlusion, lighting variations, and signer differences. However, these models have limitations, such as a restricted ability to capture complex, higher-order uncertainties involving multiple variables. They also introduce computational overhead, which can hinder real-time performance, and may not generalize well with highly variable data compared to more advanced probabilistic models [22].

The study presented in Ref. [26] introduces a bidirectional translation framework for Arabic Sign Language (ArSL) to improve communication between deaf and hearing individuals. The framework consists of two modules: one that translates Arabic sign images into text using various transfer learning models and another that converts text into sign language images. We developed a prototype using pre-trained convolutional neural networks (CNNs) such as DenseNet121, ResNet152, MobileNetV2, Xception, InceptionV3, NASNetLarge, VGG19, and VGG16. A novel fuzzy string matching score method was used to match input text with the corresponding sign language images. The dataset, featuring 7030 images across 14 classes, was collected locally from both deaf and hearing individuals. Experiments were conducted using this dataset and the CNN models.

In Ref. [27], the authors suggested an image-based method for sign language recognition using a four-phase framework. The first phase involves image processing techniques to smooth images, with skin color detection and the Viola–Jones algorithm used to segment the face and hand. In the second phase, distance and similarity features are extracted using distance count and correlation coefficient methods. The third phase classifies and identifies signs using a Neuro-Fuzzy classification algorithm. The final phase employs Natural Language Processing (NLP) to display the recognized word. The framework, implemented in MATLAB R2022a, was tested on 20 videos (100 images) of family relation signs, achieving 95% accuracy. Table 1 compares between Type-1 Neutrosophic Logic (T1NL) and Uncertainty-Aware Deep Neural Networks (UADNNs) for sign language processing [22,28,29]. Also, Table 2 provides a comparison illustrating how the proposed T2NHMM for SLR distinguishes itself from other uncertainty-handling techniques, including traditional fuzzy logic, probability-based approaches, and Type-1 Neutrosophic Systems, particularly in terms of uncertainty representation, adaptability, and real-time applicability. Finally, Table 3 summarizes the different approaches used in SLR based on the methods discussed.

### 2.1. Research Gap

The research gap between the current methods and high-level uncertainty models for SLR lies in the ability to effectively handle both epistemic (model) and Aleatoric (data) uncertainties while maintaining scalability, generalizability, and real-time performance. The current approaches, such as Gaussian Processes (GPs), Bayesian Neural Networks (BNNs), and ensemble methods, provide uncertainty quantification but struggle with computational efficiency, especially in large-scale or high-dimensional SLR datasets. These models often require complex inference techniques, making them impractical for real-time applications. On the other hand, uncertainty models like Type-1 Neutrosophic Logic and deep evidential regression offer some promise in handling noisy or ambiguous sign data but are limited in capturing higher-order uncertainties. The gap exists in developing models that not only quantify uncertainty effectively but also scale well across diverse signing styles, languages, and real-world conditions, ensuring that SLR systems are both robust and efficient.

The proposed T2NHMM for SLR fundamentally differs from traditional machine learning and deep learning approaches by focusing on explicitly modeling and managing uncertainty through a framework of truth, falsity, and indeterminacy, represented by the FOU. Unlike conventional approaches that focus on feature extraction and error minimization without directly addressing uncertainty, this model captures a range for each uncertainty level, enabling precise confidence in gesture recognition. Its interval-based ambiguity handling allows it to adapt to variations in signing styles, lighting, and occlusions, making it more flexible and less dependent on large labeled datasets. Additionally, T2NHMM inherently manages model drift, reducing the need for frequent retraining in dynamic environments. Despite requiring significant computation, its focus on uncertainty intervals offers a computationally efficient alternative to deep learning, enhancing real-time applicability in SLR with limited resources. Furthermore, T2NHMM for SLR differentiates itself from traditional fuzzy logic and probability-based methods by providing a more nuanced approach to uncertainty. While traditional fuzzy models rely on single membership degrees and probability-based methods on fixed confidence intervals, the Type-2 Neutrosophic HMM employs a three-part framework (truth, falsity, and indeterminacy) with the FOU to represent uncertainty as intervals, capturing both first- and second-order uncertainties.

### 2.2. The Need to Extend the Related Work

Type-1 Neutrosophic Hidden Markov Models (T1-NHMMs) face several challenges in sign language recognition (SLR). First, T1-NHMMs use single-valued membership functions to represent truth, indeterminacy, and falsity, which limits their ability to capture complex, higher-order uncertainties present in sign language data. This can result in inadequate handling of nuanced variations in signing styles, lighting conditions, and ambiguous gestures. Additionally, the fixed nature of T1-NHMMs makes them less adaptable to diverse scenarios, leading to difficulties in managing the wide range of uncertainties found in real-world sign language data. Furthermore, T1-NHMMs may struggle with high-dimensional data, as their static uncertainty representations can fail to capture the intricate relationships between features in complex sign language sequences, potentially leading to reduced accuracy and performance.

Type-2 Neutrosophic Hidden Markov Models (T2-NHMMs) address these limitations by using Type-2 neutrosophic sets, which incorporate membership functions with a range of possible values rather than fixed ones. This allows T2-NHMMs to model uncertainties more comprehensively, capturing complex, higher-order uncertainties and interactions between multiple variables. The flexibility of Type-2 fuzzy logic enables T2-NHMMs to better represent varying degrees of truth, indeterminacy, and falsity, making them more adaptable to the diverse and dynamic nature of sign language data. Additionally, T2-NHMMs utilize the Footprint of Uncertainty (FOU), which provides a dynamic representation of uncertainty, allowing the model to effectively manage noisy and ambiguous data. This enhanced capability improves the model’s robustness and accuracy, even in high-dimensional settings where traditional T1-NHMMs may fall short. Overall, T2-NHMMs offer a more sophisticated approach to handling the complexities of sign language recognition, leading to better performance and adaptability in real-world applications.

## 3. Methodology

The problem of subject-independent sign language recognition (SISLR) aims to build a system that accurately recognizes sign language gestures regardless of who performs the gesture. This problem is challenging due to the wide variability in individuals’ hand shapes, sizes, movement styles, and speeds. Let $X\in R^{F}$ represent a sequence of features extracted from the gesture image and F represent the number of features per gesture. $Y\in \left\{1,2,3,\ldots .C\right\}$ represents the gesture class label, where C is the total number of gesture classes. The task is to learn a function $f:X\in R^{F}\to$ $\left\{1,2,3,\ldots .C\right\}$ such that for a new gesture X, the function f(X) predicts the correct class label Y [4,5,6]. A key challenge in SISLR is the variability in signing styles. This introduces two types of uncertainty: model uncertainty (epistemic uncertainty) $u_{m}\left(X\right)$ arising from the model’s lack of knowledge about new signers it has not seen before and data uncertainty (Aleatoric uncertainty) $u_{d}\left(X\right)$ due to inherent noise in the data (e.g., gesture image quality, lighting, occlusion). Thus, the model prediction is defined as

(1) $$\hat{Y}=\arg\underset{c\in \left\{1,2,\ldots .,C\right\}}{\max}P\left(Y=c|X\right)-\left(u_{m}\left(X\right)+u_{d}\left(X\right)\right),$$

$P\left(Y=c|X\right)$ is the probability of the class label C given the input sequence X; $u_{m}\left(X\right)$ and $u_{d}\left(X\right)$ reduce the confidence of the model based on uncertainties.

**Figure 3:**
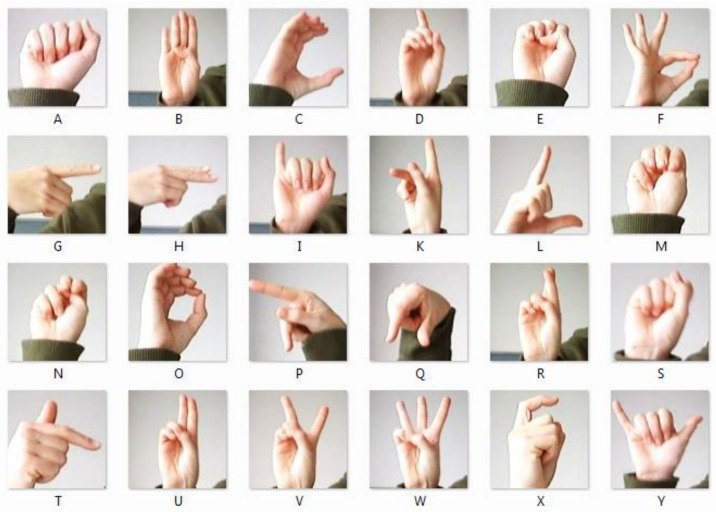
Some English alphabet in American Sign Language.

Let X consist of the following components (Kinematic Model): Hand positions: $P=\left(x,y,z\right)$, where x,y,z are the hand coordinates. Hand orientation: $O=\left(\theta ,\;\emptyset \right)$, where θ, ∅ are the angles representing hand orientation. Key joint positions: $J\in R^{n\times 3}$, where n is the number of key joints tracked on the hand. Thus, the feature vector can be represented as $X=\left[P,O,J\right]$ for each gesture. Figure 3 depicts the American Sign Language (ASL) alphabet represented through hand gestures. Each letter of the alphabet, from A to Y, is demonstrated using distinct hand shapes and orientations, showcasing the manual alphabet system used in ASL. In addition, Table 4 lists sample of hand signs along with their position, orientation, and the positions of 5 key joints in 3D coordinates. In this table, the key joints could correspond to common points on the hand like the wrist, knuckles, and fingertips. The specific joints correspond to important anatomical points of the hand in ASL gestures.

In our case, MediaPipe, developed by Google https://ai.google.dev/edge/mediapipe/solutions/guide (1 June 2024), is utilized as it is a versatile framework designed for real-time hand tracking and 3D hand pose estimation from 2D images or video streams. It features a comprehensive hand model capable of detecting key joint positions and orientations in 3D space, making it ideal for applications like hand tracking and pose estimation. Known for its speed and high optimization, MediaPipe excels at providing both 2D and 3D keypoint detection, making it a powerful tool for analyzing hand gestures efficiently.

Given a training dataset $D={\left\{\left(X_{i},Y_{i}\right)\right\}}_{i=1}^{N}$}, where N is the number of training samples, the objective is to train a model that maps input sequences to class labels by minimizing a loss function L(φ) over model parameters φ. A common choice is the categorical cross-entropy loss, where $1\left(Y_{i}=c\right)$ is an indicator function that is 1 if the true label is c, and 0 otherwise.

(2) $$L\left(\varphi \right)=\frac{1}{N}\sum_{i=1}^{N}\sum_{c=1}^{C}1\left(Y_{i}=c\right)\;log\;P\left(Y_{i}=c|X_{i};\varphi \right),$$

To improve robustness, especially in the context of subject-independent recognition, uncertainty can be explicitly modeled as part of the loss function [21]:

(3) $$L_{uncertainty}\left(\varphi \right)=L\left(\varphi \right)+\lambda \sum_{i=1}^{N}\left(u_{m}\left(X_{i}\right)+u_{d}\left(X_{i}\right)\right),$$

where λ is a regularization parameter that balances the contribution of uncertainty to the overall loss. To handle this, we propose an enhanced HMM-based SISLR framework where features are represented using Type-2 Neutrosophic Logic, which models uncertainty through truth (*T*), indeterminacy (*I*), and falsity (*F*) values. Let $X=\left\{X_{1},X_{2},\;\ldots \ldots ,\;X_{T}\right\}$ be the observed sequence of feature vectors over time *T*, $X_{t}\in R^{F}$ and $S=\left\{s_{1},s_{2},\;\ldots \ldots ,\;s_{k}\right\}$ represent the set of hidden states of the HMM, where *K* is the number of hidden states. Each feature $X_{t,i}$ in the sequence is represented as a Type-2 neutrosophic set:

(4) $$X_{t,i}=\langle T_{X_{t,i}},I_{X_{t,i}},F_{X_{t,i}}\rangle ,$$

$T_{X_{t,i}}$ represents the truth membership value of feature i at time t, $I_{X_{t,i}}$ represents the indeterminacy membership value, and $F_{X_{t,i}}$ represents the falsity membership value.

In the HMM framework, we define the following parameters: (1) Transition probabilities $A=\left(a_{ij}\right)$, where $a_{ij}=P\left(S_{j}|S_{i}\right)$ is the probability of transitioning from state $S_{i}$ to state $S_{j}$. (2) Emission probabilities $B=\left(b_{j}\left(X_{t}\right)\right)$, where $b_{j}\left(X_{t}\right)=P\left(X_{t}|S_{j}\right)$ is the probability of observing the feature vector $X_{t}$ given the hidden state $S_{j}$. (3) Initial state probabilities $\pi =\left\{\pi_{i}\right\}$, where $\pi_{i}=P(S_{i}$) is the probability of starting in state $S_{i}$ [30]. For each hidden state $S_{j}$, the emission probability $b_{j}\left(X_{t}\right)$ is modeled using the neutrosophic membership functions. Given that each feature vector $X_{t}$ is a Type-2 neutrosophic set, the emission probability is computed based on the truth, indeterminacy, and falsity membership functions for each feature at time *t*. The overall neutrosophic emission probability for a feature vector $X_{t}$ at state $S_{j}$ is defined as

(5) $$b_{j}\left(X_{t}\right)=\frac{1}{F}\sum_{i=1}^{F}\left[w_{T}T_{X_{t,i}}\left(S_{j}\right)+w_{I}I_{X_{t,i}}\left(S_{j}\right)+w_{F}F_{X_{t,i}}\left(S_{j}\right)\right],$$

$w_{T}$,$w_{I}$,$w_{F}$ are the weights associated with truth, indeterminacy, and falsity, respectively (these can be tuned based on the degree of importance of each component), and $T_{X_{t,i}}\left(S_{j}\right)$, $I_{X_{t,i}}\left(S_{j}\right)$, and $F_{X_{t,i}}\left(S_{j}\right)$ represent the truth, indeterminacy, and falsity values of feature *i* at state $S_{j}$. The likelihood of observing the sequence $X=\left\{X_{1},X_{2},\;\ldots \ldots ,\;X_{T}\right\}$ given an HMM $\lambda_{y}=\left(A_{Y},B_{Y},\pi_{Y}\right)$ for a gesture class Y, considering neutrosophic uncertainty, is computed using the neutrosophic forward algorithm:

(6) $$P\left(X|\lambda_{y}\right)=\sum_{s_{1},s_{2},\;\ldots \ldots ,\;s_{k}}\pi_{Y,S_{1}}\prod_{t=1}^{T}a_{Y,S_{t-1},S_{t}}b_{Y,S_{t}}\left(X_{t}\right),$$

where the emission probabilities $b_{Y,S_{t}}\left(X_{t}\right)$ are computed using Type-2 Neutrosophic Logic.

For classification, the observed sequence $X=\left\{X_{1},X_{2},\;\ldots \ldots ,\;X_{T}\right\}$ is compared to the model for each gesture class Y. The neutrosophic similarity measure between the observed sequence and each model is computed as

(7) $$S\left(X,\lambda_{y}\right)=\sum_{t=1}^{T}\sum_{i=1}^{F}\left[\frac{T_{X_{t,i}}\cap \;T_{R_{Y,t,i}}+I_{X_{t,i}}\cap \;I_{R_{Y,t,i}}+F_{X_{t,i}}\cap \;F_{R_{Y,t,i}}}{T_{X_{t,i}}\cup \;T_{R_{Y,t,i}}+I_{X_{t,i}}\cup \;I_{R_{Y,t,i}}+F_{X_{t,i}}\cup \;F_{R_{Y,t,i}}}\right]$$

$R_{Y,t,i}$ is the reference feature vector for gesture class Y at time t, and ∩ and ∪ represent the intersection and union of the FOU for the truth, indeterminacy, and falsity membership values. The recognized gesture $\hat{Y}$ is the one with the highest neutrosophic similarity score:

(8) $$\hat{Y}=\arg\underset{Y\in \left\{1,2,\ldots ..,C\right\}}{\max}S\left(X,\lambda_{y}\right)$$

Training the HMM involves estimating the parameters $\lambda_{y}=\left(A_{Y},B_{Y},\pi_{Y}\right)$ for each gesture class Y using the Baum–Welch algorithm, an extension of the Expectation–Maximization (EM) algorithm for HMMs. During the training, the emission probabilities are computed using the neutrosophic membership functions, allowing the model to handle the inherent uncertainty in the observed features. See [18,22,31,32,33] for more details.

**Figure 4:**
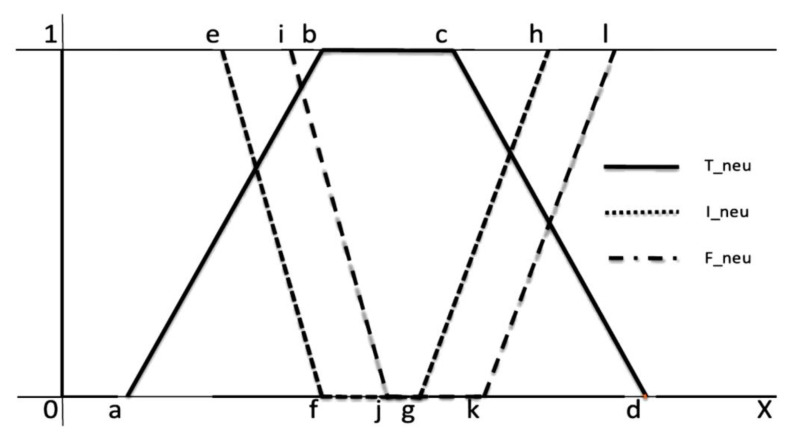
Linear trapezoidal neutrosophic membership function.

T2NL is an advanced framework for dealing with uncertainty, imprecision, vagueness, and inconsistency. This approach provides a more flexible and precise method for handling higher levels of uncertainty and complex information. A neutrosophic set is characterized by three functions: truth membership (*T*), indeterminacy membership (*I*), and falsity membership (*F*). In T2NL, these functions are not single values but ranges (intervals). Truth (*T*) represents the degree to which an element is true. In Type-2 Neutrosophic Logic, it is an interval $\left[T_{L},T_{U}\right],0\leq T_{L}\leq T_{U}\leq 1$. Indeterminacy (*I*) represents the degree of indeterminacy or uncertainty. It is an interval $\left[I_{L},I_{U}\right],0\leq I_{L}\leq I_{U}\leq 1$. Falsity (*F*) represents the degree to which an element is false. It is an interval $\left[F_{L},F_{U}\right],0\leq F_{L}\leq F_{U}\leq 1$. A Type-2 neutrosophic set *A* in a universe of discourse *X* is defined as $A=\left\{\langle x,\left(F\left(x\right),I\left(x\right),F(x)\right)\rangle :x\in X\right\},\forall x\in X,T\left(x\right)=\left[T_{L}\left(x\right),T_{U}\left(x\right)\right],I\left(X\right)=\left[I_{L}\left(x\right),I_{U}\left(x\right)\right],F\left(X\right)=\left[F_{L}\left(x\right),F_{U}\left(x\right)\right]$. In our case, a linear trapezoidal neutrosophic number, as illustrated in Figure 4, is used that is defined as [32]

(9) $${Ã}_{Neu}=(a,b,c,d;e,f,g,h;i,j,k,l)$$

**Figure 5:**
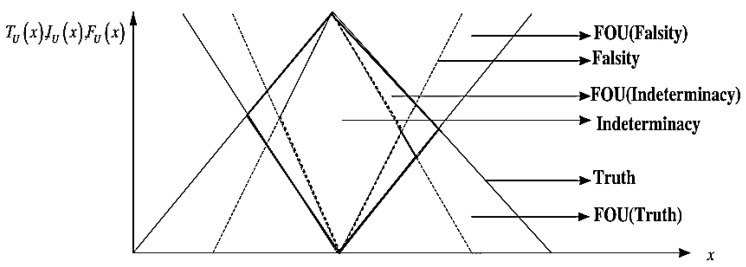
Graphical representation of Type-2 neutrosophic membership function [8].

The truth, indeterminacy, and falsity membership is defined as [34]

(10) $$Truth\;membership\;function=T_{Ã\_Neu}=\left\{\begin{array}{c} \begin{array}{cc} 0 & \;\;\;\;\;\;\;\;\;\;\;\;\;\;\;\;\;\;x<a \end{array}\\ \begin{array}{cc} \frac{x-a}{b-a} & \;\;\;\;\;\;\;\;\;\;\;a\leq x<b \end{array}\\ \begin{array}{c} \begin{array}{cc} 1 & \;\;\;\;\;\;\;\;\;\;\;\;\;\;\;b\leq x<c \end{array}\\ \begin{array}{c} \begin{array}{cc} \frac{d-x}{d-c} & \;\;\;\;\;\;\;\;\;\;\;\;c\leq x\leq d \end{array}\\ \begin{array}{cc} 0 & \;\;\;\;\;\;\;\;\;\;\;\;\;\;\;\;\;\;\;x>d \end{array} \end{array} \end{array} \end{array}\right.$$

(11) $$Indeterminacy\;membership\;function=I_{Ã\_Neu}=\left\{\begin{array}{c} \begin{array}{cc} 1 & \;\;\;\;\;\;\;\;\;\;\;\;\;x<e \end{array}\\ \begin{array}{cc} \frac{f-x}{f-e} & \;\;\;\;\;\;\;\;e\leq x<f \end{array}\\ \begin{array}{c} \begin{array}{cc} 0 & \;\;\;\;\;\;\;\;\;\;\;f\leq x<g \end{array}\\ \begin{array}{c} \begin{array}{cc} \frac{x-g}{h-g} & \;\;\;\;\;\;g\leq x\leq h \end{array}\\ \begin{array}{cc} 1 & \;\;\;\;\;\;\;\;\;\;\;\;\;x>h \end{array} \end{array} \end{array} \end{array}\right.$$

(12) $$Falsity\;membership\;function=F_{Ã\_Neu}=\left\{\begin{array}{c} \begin{array}{cc} 1 & \;\;\;\;\;\;\;\;\;\;\;\;\;x<i \end{array}\\ \begin{array}{cc} \frac{j-x}{j-i} & \;\;\;\;\;\;\;\;\;\;\;i\leq x<j \end{array}\\ \begin{array}{c} \begin{array}{cc} 0 & \;\;\;\;\;\;\;\;\;\;\;\;\;\;\;j<x<k \end{array}\\ \begin{array}{c} \begin{array}{cc} \frac{x-k}{l-k} & \;\;\;\;\;\;\;\;\;\;k\leq x\leq l \end{array}\\ \begin{array}{cc} 1 & \;\;\;\;\;\;\;\;\;\;\;\;\;\;\;\;x>l \end{array} \end{array} \end{array} \end{array}\right.$$

where ${0\leq T}_{{Ã}_{Neu}}\left(x\right)+I_{{Ã}_{Neu}}(x)+I_{{Ã}_{Neu}}(x)\leq 3,x\in {Ã}_{Neu}$. In general, linear trapezoidal functions are computationally simpler and more efficient to implement compared to more complex functions like Gaussian or other non-linear forms. The trapezoidal shape is defined by four parameters, making it straightforward to calculate the membership values, which is essential for high-performance systems that need to process gesture inputs continuously and respond without delay. Furthermore, with its two flat regions (low and high membership), it is effective at modeling indeterminate areas where uncertainty is high, offering more granularity in regions that are neither fully true nor false, aligning with the characteristics of sign language recognition systems [33]. Figure 5 depicts the graphical representation of FOU for every membership function *T*, *I*, and *F.*

In Type-2 Neutrosophic Logic, the FOU is a critical concept used to model uncertainty by capturing the range of possible values for each membership function. Each membership function is represented by a fuzzy set, and the FOU is defined for each as the region between its upper and lower membership functions. The FOU for each membership function forms a band-like region representing the uncertainty in the specific membership degree. The lower bound reflects conservative or minimal estimations (most certain values), while the upper bound represents optimistic or maximal estimations (least certain values). The width of the FOU indicates the degree of uncertainty, with wider FOUs signifying higher variability. The FOU for each membership function captures data variability and modeling uncertainty, enables flexible similarity measures by accounting for overlapping FOUs, and supports decision-making by balancing truth, indeterminacy, and falsity in the presence of real-world imprecision [8,9].

Membership functions map data points to degrees of truth, indicating how strongly a feature belongs to a fuzzy set. For instance, for modeling the “height” of a person, a trapezoidal membership function might have “Low”, “Medium”, and “High” regions, with overlap between them to handle ambiguity. A membership function in Type-2 Neutrosophic Logic quantifies the degrees of truth, indeterminacy, and falsity for an element, using ranges to capture uncertainty rather than fixed values. This method models variability and ambiguity by defining possible values within a band-like region, using the FOU. When applied to hand sign features in sign language recognition, the membership function evaluates characteristics like finger angles, hand position, and motion trajectories. By linking these features to degrees of truth, indeterminacy, and falsity, the model accounts for variations in signing styles, incomplete gestures, or environmental noise, enabling more robust and adaptive recognition across diverse contexts.

Formally, a single-valued neutrosophic set (SVNS) $\overset{˘}{S}$ on universal set *U* is characterized by truth membership function $TMF\left(\emptyset_{\overset{˘}{S}}\right)$, indeterminacy membership function $IMF\left(\psi_{\overset{˘}{S}}\right),$ and falsity membership function $FMF\left(\varphi_{\overset{˘}{S}}\right)$, respectively, in the following way [10,11]:

(13) $$\overset{˘}{S}=\left\{\langle \xi ,\left(\emptyset_{\overset{˘}{S}}\left(\xi \right),\psi_{\overset{˘}{S}}\left(\xi \right),\varphi_{\overset{˘}{S}}\left(\xi \right)\right)\rangle :\xi \in U,\emptyset_{\overset{˘}{S}}\left(\xi \right),\psi_{\overset{˘}{S}}\left(\xi \right),\varphi_{\overset{˘}{S}}\left(\xi \right)\in \left[0,1\right]\right\}$$

(14) $$\mathrm{Such}\;\mathrm{that}\;{0\leq \emptyset }_{\overset{˘}{S}}\left(\xi \right),\psi_{\overset{˘}{S}}\left(\xi \right),\varphi_{\overset{˘}{S}}\left(\xi \right)\leq 3.$$

**Figure 6:**
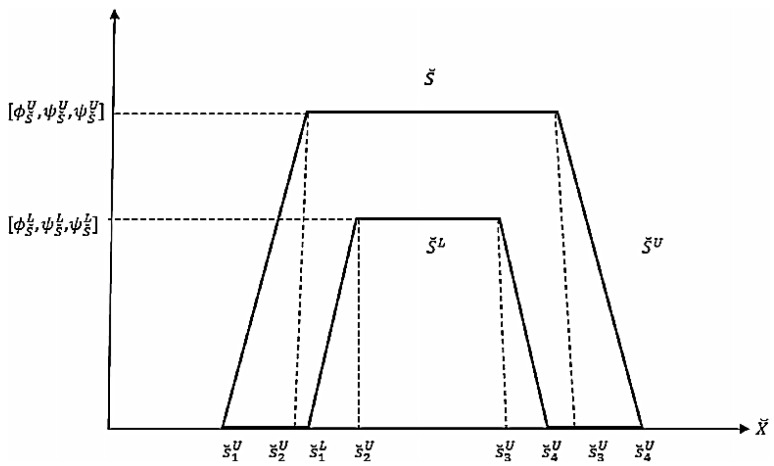
An interval Type-2 trapezoidal neutrosophic number [8].

Let $\overset{˘}{S}\left(\xi \right)=\left[{\overset{˘}{S}}^{U}\left(\xi \right),{\overset{˘}{S}}^{L}\left(\xi \right)\right]$ be an interval Type-2 neutrosophic set (IT2NS) on universal set U, where ξ∈U and ${\overset{˘}{S}}^{U}:U\to \left[0,1\right]$ and ${\overset{˘}{S}}^{L}:U\to \left[0,1\right]$ are two Type-1 neutrosophic sets (T1NSs) known as upper and lower neutrosophic sets, respectively, having the condition $0\leq {\overset{˘}{S}}^{L}\left(\xi \right)\leq {\overset{˘}{S}}^{U}\left(\xi \right)\leq 1$ defined as follows [19]:

(15) $$\overset{˘}{S}=\left\{\langle \xi ,\left(\left[{\emptyset_{\overset{˘}{S}}}^{U}\left(\xi \right),{\emptyset_{\overset{˘}{S}}}^{L}\left(\xi \right)\right],\left[{\psi_{\overset{˘}{S}}}^{U}\left(\xi \right),{\psi_{\overset{˘}{S}}}^{L}\left(\xi \right)\right],\left[{\varphi_{\overset{˘}{S}}}^{U}\left(\xi \right),{\varphi_{\overset{˘}{S}}}^{L}\left(\xi \right)\right]\right)\rangle :\xi \in U\right\}$$

(16) $$\left[{\emptyset_{\overset{˘}{S}}}^{U}\left(\xi \right),{\emptyset_{\overset{˘}{S}}}^{L}\left(\xi \right)\right],\left[{\psi_{\overset{˘}{S}}}^{U}\left(\xi \right),{\psi_{\overset{˘}{S}}}^{L}\left(\xi \right)\right],\left[{\varphi_{\overset{˘}{S}}}^{U}\left(\xi \right),{\varphi_{\overset{˘}{S}}}^{L}\left(\xi \right)\right]\in \left[0,1\right]$$

(17) $$\emptyset_{\overset{˘}{S}}\left(\xi \right)=\left\{\begin{array}{cc} \frac{\left(\xi -{\overset{˘}{S}}_{1}\right)\emptyset_{\overset{˘}{S}}}{{\overset{˘}{S}}_{2}-{\overset{˘}{S}}_{1}} & {\overset{˘}{S}}_{1}\leq \xi \leq {\overset{˘}{S}}_{2}\\ \emptyset_{\overset{˘}{S}} & {\overset{˘}{S}}_{2}\leq \xi \leq {\overset{˘}{S}}_{3}\\ \begin{array}{c} \frac{\left({\overset{˘}{S}}_{4}-\xi \right)\emptyset_{\overset{˘}{S}}}{{\overset{˘}{S}}_{4}-{\overset{˘}{S}}_{3}}\\ 0 \end{array} & \begin{array}{c} {\overset{˘}{S}}_{3}\leq \xi \leq {\overset{˘}{S}}_{4}\\ otherwise \end{array} \end{array}\right.$$

(18) $$\psi_{\overset{˘}{S}}\left(\xi \right)=\left\{\begin{array}{cc} \frac{{\overset{˘}{S}}_{2}-\xi +\left(\xi -{\overset{˘}{S}}_{1}\right)\psi_{\overset{˘}{S}}}{{\overset{˘}{S}}_{2}-{\overset{˘}{S}}_{1}} & {\overset{˘}{S}}_{1}\leq \xi \leq {\overset{˘}{S}}_{2}\\ \psi_{\overset{˘}{S}} & {\overset{˘}{S}}_{2}\leq \xi \leq {\overset{˘}{S}}_{3}\\ \begin{array}{c} \frac{\xi -{\overset{˘}{S}}_{3}+\left({\overset{˘}{S}}_{4}-\xi \right)\psi_{\overset{˘}{S}}}{{\overset{˘}{S}}_{4}-{\overset{˘}{S}}_{3}}\\ 1 \end{array} & \begin{array}{c} {\overset{˘}{S}}_{3}\leq \xi \leq {\overset{˘}{S}}_{4}\\ otherwise \end{array} \end{array}\right.$$

(19) $$\varphi_{\overset{˘}{S}}\left(\xi \right)=\left\{\begin{array}{cc} \frac{{\overset{˘}{S}}_{2}-\xi +\left(\xi -{\overset{˘}{S}}_{1}\right)\varphi_{\overset{˘}{S}}}{{\overset{˘}{S}}_{2}-{\overset{˘}{S}}_{1}} & {\overset{˘}{S}}_{1}\leq \xi \leq {\overset{˘}{S}}_{2}\\ \varphi_{\overset{˘}{S}} & {\overset{˘}{S}}_{2}\leq \xi \leq {\overset{˘}{S}}_{3}\\ \begin{array}{c} \frac{\xi -{\overset{˘}{S}}_{3}+\left({\overset{˘}{S}}_{4}-\xi \right)\varphi_{\overset{˘}{S}}}{{\overset{˘}{S}}_{4}-{\overset{˘}{S}}_{3}}\\ 1 \end{array} & \begin{array}{c} {\overset{˘}{S}}_{3}\leq \xi \leq {\overset{˘}{S}}_{4}\\ otherwise \end{array} \end{array}\right.$$

where $\emptyset_{\overset{˘}{S}}=\left[{\emptyset_{\overset{˘}{S}}}^{U},{\emptyset_{\overset{˘}{S}}}^{L}\right],\psi_{\overset{˘}{S}}=\left[{\psi_{\overset{˘}{S}}}^{U},{\psi_{\overset{˘}{S}}}^{L}\right]and{\;\varphi }_{\overset{˘}{S}}=\left[{\varphi_{\overset{˘}{S}}}^{U},{\varphi_{\overset{˘}{S}}}^{L}\right]$ are interval Type-2 neutrosophic numbers (IT2NNs). The number $\overset{˘}{S}$ can be represented as (see Figure 6)

(20) $$\overset{˘}{S}=\left[{\overset{˘}{S}}^{U},{\overset{˘}{S}}^{L}\right]=\left[\left({\overset{˘}{S}}_{1}^{U},{\overset{˘}{S}}_{2}^{U},{\overset{˘}{S}}_{3}^{U},{\overset{˘}{S}}_{4}^{U};\emptyset_{\overset{˘}{S}}^{U},\psi_{\overset{˘}{S}}^{U},\varphi_{\overset{˘}{S}}^{U}\right),\left({\overset{˘}{S}}_{1}^{L},{\overset{˘}{S}}_{2}^{L},{\overset{˘}{S}}_{3}^{L},{\overset{˘}{S}}_{4}^{L};\emptyset_{\overset{˘}{S}}^{L},\psi_{\overset{˘}{S}}^{L},\varphi_{\overset{˘}{S}}^{L}\right)\right]$$

This is called interval Type-2 trapezoidal neutrosophic logic number (IT2TrNN), where

(21) $${0\leq \overset{˘}{S}}_{1}^{U}\leq {\overset{˘}{S}}_{2}^{U}\leq {\overset{˘}{S}}_{3}^{U}\leq {\overset{˘}{S}}_{4}^{U}\leq 1,{0\leq \overset{˘}{S}}_{1}^{L}\leq {\overset{˘}{S}}_{2}^{L}\leq {\overset{˘}{S}}_{3}^{L}\leq {\overset{˘}{S}}_{4}^{L}\leq 1$$

(22) $${0\leq \emptyset }_{\overset{˘}{S}}^{L}\leq \emptyset_{\overset{˘}{S}}^{U}\leq 1,{0\leq \psi }_{\overset{˘}{S}}^{L}\leq \psi_{\overset{˘}{S}}^{U}\leq 1,{0\leq \varphi }_{\overset{˘}{S}}^{L}\leq \varphi_{\overset{˘}{S}}^{U}\leq 1$$

### 3.1. Step-by-Step Approach to Integrating Type-2 Neutrosophic Logic in HMM

- Neutrosophic Feature Extraction: Each gesture’s visual or motion features are transformed into Type-2 neutrosophic sets, representing truth (*T*), indeterminacy (*I*), and falsity (*F*), with a degree of uncertainty each defined by upper and lower bounds. These features are then passed to the HMM as part of the observation data. For observation $O_{t}$, we represent it as
  (23) $${N(O}_{t})=\langle \langle T_{lower}\left(O_{t}\right),T_{upper}\left(O_{t}\right)\rangle ,\langle I_{lower}\left(O_{t}\right),I_{upper}\left(O_{t}\right)\rangle ,\langle F_{lower}\left(O_{t}\right),F_{upper}\left(O_{t}\right)\rangle \rangle$$
  ${T(O}_{t}),\;{I(O}_{t})$ and ${F(O}_{t})$ capture the truth, indeterminacy, and falsity of observation ${\;O}_{t}$, and $T_{lower}\left(O_{t}\right)$ and $T_{upper}\left(O_{t}\right)$ represent the lower and upper bounds for truth membership (similarly for indeterminacy and falsity).
- Observation Probability in HMM (Adjusted with Neutrosophic Sets): The observation probabilities in HMM, which traditionally map features to states, are modified to include the neutrosophic membership functions for truth, indeterminacy, and falsity, providing more flexible modeling of noisy or uncertain gestures. In a standard HMM, the observation probability ${B_{j}(O}_{t})$ at state *j* is the likelihood of observing feature $O_{t}$ in that state. With the neutrosophic framework, the observation probability is adjusted to include neutrosophic membership values:
  (24) $${B_{j}(O}_{t})=\propto \times {T(O}_{t})+\beta \times {I(O}_{t})+\gamma {\times F(O}_{t})$$
  ∝, β, and γ are weight parameters that balance the contribution of truth, indeterminacy, and falsity to the observation probability. This formula adjusts the likelihood of each observation based on its degree of certainty or uncertainty as captured by the neutrosophic sets.
- Forward Probability Using Neutrosophic HMM: The forward algorithm calculates the probability of observing a sequence up to time *t* and being in state *j* (forward probability $\alpha_{t}(j)$. For the neutrosophic HMM, the forward probability is updated using the neutrosophic-adjusted observation probabilities:
  (25) $$\alpha_{t}\left(j\right)=\left(\sum_{t=1}^{N}\alpha_{t-1}\left(i\right)A_{ij}\right)\times {B_{j}(O}_{t})$$
  $\alpha_{t}\left(j\right)$ is the forward probability at time *t* for state *j*, $A_{ij}$ is the transition probability from state *i* to state *j*, and ${B_{j}(O}_{t})$ is the neutrosophic-adjusted observation probability for state *j* at time *t*. The forward algorithm now integrates uncertainty into the observation step through the Type-2 neutrosophic sets.
- Backward Probability with Neutrosophic HMM: Similarly, the backward probability $\beta_{t}\left(j\right)$, which represents the probability of observing the sequence from time *t* + 1 to the end given state *i* at time *t*, is also updated:
  (26) $$\beta_{t}\left(j\right)=\left(\sum_{t=1}^{N}A_{ij}\times {B_{j}(O}_{t+1})\right)\times \beta_{t+1}\left(j\right)$$
  ${B_{j}(O}_{t+1})$ is again adjusted by the neutrosophic membership functions.
- Baum–Welch algorithm for Neutrosophic HMM Training: The Baum–Welch algorithm, used for training HMMs, can also be adapted to the neutrosophic HMM. In each iteration, the algorithm updates the model parameters (transition probabilities A and observation probabilities *B*) to maximize the likelihood of the observation sequence. The new E-step (expectation step) uses the neutrosophic-adjusted forward and backward probabilities, while the M-step (maximization step) remains the same but operates with neutrosophic-adjusted observation probabilities.
  ▪ E-step (Expectation): Compute the neutrosophic forward and backward probabilities using Equation (24).
  ▪ M-step (Maximization): Update the transition and emission probabilities using the neutrosophic-enhanced likelihood estimates.
- Final Recognition and Decision-Making: Once the HMM is trained with neutrosophic observations, the final step is to perform gesture recognition. For a given observation sequence, the HMM will compute the most likely state sequence (corresponding to gestures) using the Viterbi algorithm, which has been modified to incorporate neutrosophic-adjusted probabilities. The recognition decision will be influenced by both the temporal sequence (modeled by HMM) and the uncertainty handling (modeled by Type-2 Neutrosophic Logic), ensuring a more robust and accurate interpretation of sign language gestures.

### 3.2. Algorithm: Type-2 Neutrosophic HMM for Hand Recognition

Here is an algorithm for a Type-2 Neutrosophic Hidden Markov Model (HMM)-based hand recognition system that integrates Type-2 Neutrosophic Logic for managing uncertainty.

**Input:**

- Observed sequence of hand gesture features $O=\left(o_{1},o_{2},\ldots ..,o_{T}\right)$
- State transition probabilities $A=\left\{a_{ij}\right\}$, modeled as Type-2 neutrosophic sets.
- Observation probabilities $B=\left\{b_{j}(o)\right\}$, modeled as Type-2 neutrosophic sets.
- Initial state distribution $\pi =\left\{\pi_{i}\right\}$, modeled as Type-2 neutrosophic sets.

**Output:**

- Recognized hand gestures with an associated probability (truth, indeterminacy, and falsity values).

**Step 1: Initialization**

1. Define Model Parameters:
  - Define the number of hidden states *N* and the length of the observation sequence *T*.
  - Initialize the state transition matrix $A=\left[a_{ij}\right]$, the observation likelihood matrix $B=\left[b_{j}(o)\right]$, and the initial distribution $\pi =\left\{\pi_{i}\right\}$, all as Type-2 neutrosophic sets with membership functions for truth $\mu_{T}$, indeterminacy $\mu_{I}$, and falsity $\mu_{F}$.
    (27) $$a_{ij}\left(t\right)={FOU}_{A_{ij}}\left(t\right)=\left[\pi_{A_{ij}}^{low}\left(t\right),\pi_{A_{ij}}^{High}\left(t\right)\;\right],$$
    (28) $$b_{i}\left(o_{t}\right)={FOU}_{B_{i}}\left(o_{t}\right)=\left[\pi_{B_{i}}^{low}\left(o_{t}\right),\pi_{B_{i}}^{High}\left(o_{t}\right)\right],$$
    (29) $$\pi_{i}={FOU}_{\pi_{i}}=\left[\pi_{\pi_{i}}^{low},\pi_{\pi_{i}}^{High}\;\right],$$
    ${FOU}_{A_{ij}}\left(t\right)$ denotes the range of possible transition probabilities from $s_{i}$ given observation at time *t* with $\pi_{A_{ij}}^{low}\left(t\right)$ and $\pi_{A_{ij}}^{High}\left(t\right)$ as the lower and upper membership functions. ${FOU}_{B_{i}}\left(o_{t}\right)$ presents the range of possible observation $o_{t}$ with $\pi_{B_{i}}^{low}\left(o_{t}\right)$ and $\pi_{B_{i}}^{High}\left(o_{t}\right)$ as the lower and upper membership functions. ${FOU}_{\pi_{i}}$ is the range of possible transition probabilities for state $s_{i}$ with $\pi_{\pi_{i}}^{low}$ and $\pi_{\pi_{i}}^{High}$ as the lower and upper membership functions

2. Initialize the Forward Probability Matrix: For each state *j* at time *t* = 1:
  (30) $$\alpha_{1}\left(j\right)=\langle \pi_{T}\left(\pi_{j}\right),\pi_{I}\left(\pi_{j}\right),\pi_{F}\left(\pi_{j}\right)\rangle .\langle \pi_{T}\left(b_{j}(o_{1}\right),\pi_{I}\left(b_{j}(o_{1}\right),\pi_{F}\left(b_{j}(o_{1}\right)\rangle$$

**Step 2: Forward Algorithm**

For each time step *t* = 2 to *T*, and for each state *j*:

1. Compute the Forward Probability $\alpha_{t}\left(j\right)$:
  (31) $$\alpha_{t}\left(j\right)=\sum_{i=1}^{N}\begin{array}{c} \langle \pi_{T}\left(\alpha_{t-1}\left(i\right)\right),\pi_{I}\left(\alpha_{t-1}\left(i\right)\right),\pi_{F}\left(\alpha_{t-1}\left(i\right)\right)\rangle .\langle \pi_{T}\left(a_{ij}\right){,\pi }_{I}\left(a_{ij}\right),\pi_{F}\left(a_{ij}\right)\rangle \\ .\langle \pi_{T}\left(b_{j}(o_{t}\right),\pi_{I}\left(b_{j}(o_{t}\right),\pi_{F}\left(b_{j}(o_{t}\right)\rangle \end{array}$$
  This step calculates the truth, indeterminacy, and falsity values for the forward probability by multiplying and summing the corresponding neutrosophic components.

**Step 3: Viterbi Algorithm for Optimal State Path**

1. Initialization (at *t* = 1): For each state *j*:
  (32) $$\delta_{1}\left(j\right)=\langle \pi_{T}\left(\pi_{j}\right),\pi_{I}\left(\pi_{j}\right),\pi_{F}\left(\pi_{j}\right)\rangle .\langle \pi_{T}\left(b_{j}(o_{1}\right),\pi_{I}\left(b_{j}(o_{1}\right),\pi_{F}\left(b_{j}(o_{1}\right)\rangle$$
  $\psi_{1}\left(j\right)=0$ (Backpointer initialization)

2. Recursion (for *t* = 2 to *T*): For each state *j* at time *t*:
  (33) $$\delta_{t}\left(j\right)=\underset{t}{\max}\left[\delta_{t-1}\left(i\right).\langle \pi_{T}\left(a_{ij}\right),\pi_{I}\left(a_{ij}\right),\pi_{F}\left(a_{ij}\right)\rangle \right]\;\;\;\;.\langle \pi_{T}\left(b_{j}(o_{t}\right),\pi_{I}\left(b_{j}(o_{t}\right),\pi_{F}\left(b_{j}(o_{t}\right)\rangle$$

Update the backpointer ${\;\psi }_{t}\left(j\right)$:

(34) $${\;\psi }_{t}\left(j\right)=\arg\underset{i}{\max}\left[\delta_{t-1}\left(i\right).\langle \pi_{T}\left(a_{ij}\right),\pi_{I}\left(a_{ij}\right),\pi_{F}\left(a_{ij}\right)\rangle \right]$$

3. Termination (at *t* = *T*): Find the best state at time *T*:
  (35) $$P^{*}=\underset{j}{\max}\langle \pi_{T}\left(\delta_{T}\left(j\right)\right),\pi_{I}\left(\delta_{T}\left(j\right)\right),\pi_{F}\left(\delta_{T}\left(j\right)\right)\rangle$$

Trace back the state sequence using the backpointer $\psi_{T}\left(j\right)$

**Step 4: Gesture Recognition Decision**

1. Aggregate Probabilities for the hand gesture *G*: The final probability score for hand gesture recognition is calculated by combining the forward or Viterbi probabilities:
  (36) $$P\left(G|O\right)=\langle \pi_{T}\left(P\left(G|O\right)\right),\pi_{I}\left(P\left(G|O\right)\right),\pi_{F}P\left(G|O\right)\rangle$$

This score represents the neutrosophic truth, indeterminacy, and falsity for the hand gesture based on the sequence *O.*

2. Decision Rule:
  - If $\pi_{T}\left(P\left(G|O\right)\right)$ is high, classify the gesture as *G*.
  - If $\pi_{I}\left(P\left(G|O\right)\right)$ is high, there is uncertainty in recognizing *G*, and further analysis may be required.
  - If $\pi_{F}P\left(G|O\right)$ is high, reject the classification.

**Step 5: Post-processing and Output**

Output the recognized gesture *G* along with the neutrosophic truth, indeterminacy, and falsity values, helping to interpret the confidence and uncertainty in the recognition. This algorithm can effectively handle uncertainties inherent in hand gesture recognition systems, making the model more robust to noise, ambiguity, and unseen gestures.

### 3.3. Key Parameters Influencing the Performance of Type-2 Neutrosophic HMM

The effectiveness of Type-2 Neutrosophic Hidden Markov Models (HMMs) is significantly impacted by several essential parameters that facilitate their capacity to manage uncertainty and variability in applications such as sign language recognition. Critical factors include the design of membership functions for truth, indeterminacy, and falsehood, which can alter uncertainty representation; the FOU, which determines how much variability can be accommodated; and the transition and emission probabilities that govern state changes and output observations, respectively. The quality, quantity, and diversity of training data are vital for model generalization, while noise levels can challenge the model’s accuracy in interpreting input. Furthermore, the selection of temporal features affects the model’s ability to capture dynamic gestures. Model complexity, including the number of hidden states, can influence both fit and the risk of overfitting. By carefully tuning these parameters, the performance of Type-2 Neutrosophic HMMs can be optimized for specific applications, ensuring that they effectively handle the inherent uncertainties present in the data.

The following section will assess the effectiveness of these critical parameters in influencing the performance of the proposed model. This validation will involve systematic experiments designed to quantify how variations in each parameter—such as the design of membership functions, the Footprint of Uncertainty, transition and emission probabilities, training data diversity, noise levels, and temporal features—impact the model’s accuracy, robustness, and generalization capabilities. By analyzing the results, we aim to establish a clearer understanding of how these parameters contribute to the overall efficacy of the Type-2 Neutrosophic HMM in tasks like sign language recognition.

## 4. Results and Discussions

**Figure 7:**
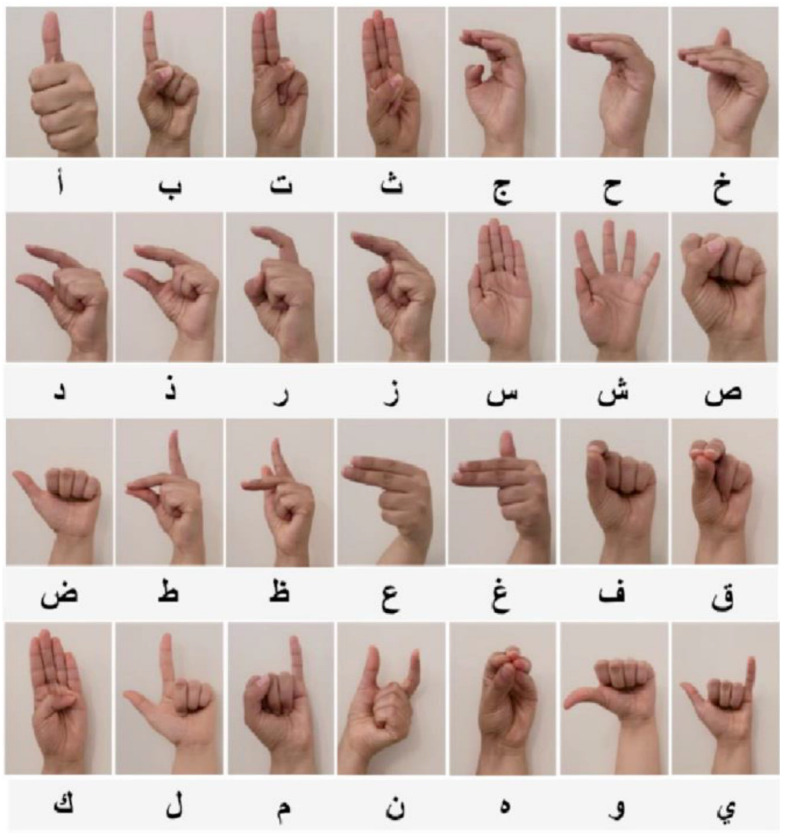
Recorded ArSL alphabet.

**Figure 8:**
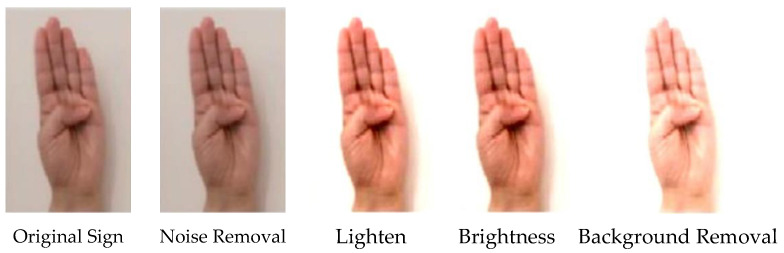
Preprocessing techniques for enhancing sign language image quality.

The accuracy of the proposed Type-2 Neutrosophic Markov Chain Model for subject-independent sign language recognition was evaluated using gesture image samples sourced from [35,36]. The Dell Inspiron N5110 laptop is manufactured by Dell Inc., a multinational company headquartered in Round Rock, Texas, USA. The benchmark dataset for subject-independent sign language recognition contains thousands of gesture samples from a large set of signers to ensure generalization across individuals. Figure 7 illustrates sample of the Arabic Sign Language (ArASL) alphabet, showcasing hand gestures corresponding to each letter in the Arabic script. Figure 8 outlines key preprocessing techniques applied to enhance the quality and clarity of sign language images, including steps such as noise removal, lightening, brightness adjustment, and background removal. These dataset often include 1000 distinct gesture signs, with each sign being performed by multiple participants 50 signers, to capture a wide range of signing variations. The number of samples per sign is 10 samples per signer for each gesture. This large dataset size supports robust training and testing, ensuring that the model can recognize signs from unseen subjects in varying conditions, such as different lighting, orientations, and hand shapes. The split into training and testing sets is carefully designed to ensure no overlap of subjects, making it suitable for subject-independent recognition tasks. Five random samples per sign were used for training, while two samples were assigned to the registered test group and the rest to the unregistered group. The recognition rate, defined as the percentage of correctly identified hand postures, served as the evaluation metric.

The system was implemented using Google Colab (Python 2.7) and tested on a Dell Inspiron N5110 (Dell Inc., Round Rock, TX, USA) with 64-bit Windows 7, 4 GB RAM, and an Intel i5-2410M CPU at 2.30 GHz. The Intel i5-2410M CPU is manufactured by Intel Corporation, a leading semiconductor company. Intel’s headquarters is located in Santa Clara, CA, USA. Our current model is proficient at recognizing individual words in sign language. However, identifying word boundaries within continuous sentence gestures presents a significant challenge. Fortunately, modern technologies have advanced considerably in handling such complexities. Python, as a versatile programming language, offers a rich ecosystem of libraries such as OpenCV, MediaPipe, TensorFlow, and PyTorch, (OpenCV-python 4.10.0.84, MediaPipe v0.10.18, TensorFlow v2.16.1, PyTorch 2.4) which facilitate gesture recognition by combining computer vision techniques with machine learning. These tools allow for real-time video processing, feature extraction, and dynamic gesture tracking, enabling models to achieve higher accuracy in recognizing both isolated signs and continuous gesture sequences.

In our case, selecting parameters for the FOU is determined empirically by analyzing the variability and distribution of features. Statistical measures like mean, standard deviation, and range are used to determine the spread of these features across samples from different individuals or repetitions of the same sign. The upper membership function (UMF) is set to encompass the full range of variability, typically extending outward by two standard deviations to include outliers and natural variations. The lower membership function (LMF) is set within one standard deviation to define the core region of high certainty. Features with higher variability require broader UMFs, while more consistent features need narrower bounds. Visualization tools such as scatter plots or histograms can aid in identifying clusters for fine-tuning these bounds, ensuring the FOU reflects both typical and uncertain signing styles.

Regarding the features’ membership functions, the shape of the membership function (e.g., triangular, trapezoidal, Gaussian) is chosen based on the data’s smoothness and distribution, with trapezoidal functions suited for linear transitions. The initial thresholds and bounds can be set empirically using data statistics like quartiles or percentiles (e.g., for a normalized range [0, 1], assign “Low” = [0, 0.25], “Medium” = [0.2, 0.8], and “High” = [0.75, 1]). These parameters can be refined by evaluating performance on a validation set and optimizing overlap regions to handle uncertainty effectively. Additionally, expert knowledge can further fine-tune boundaries to better align with real-world behavior.

Experiment 1: Comparison between Type-1 and Type-2 neutrosophic systems

**Aim:** The goal of this experiment is to evaluate and compare the performance of Type-1 Neutrosophic Markov Chain Model and Type-2 Neutrosophic Markov Chain Model in subject-independent sign language recognition, with a focus on how each model handles uncertainty, particularly in varying conditions like noise, unseen signers, and data variability.

**Procedure:** The procedure involves using the same dataset of sign language gestures for both models, ensuring subject-independent variations, different noise levels, and occlusions are included. The Type-1 and Type-2 neutrosophic models will be implemented and compared. In the Type-1 model, membership functions define crisp boundaries for truth, indeterminacy, and falsehood, whereas the Type-2 model extends these to capture uncertainty with upper and lower membership functions. The models will be evaluated based on several key metrics: accuracy (the rate of correctly classified signs), uncertainty estimation (the effectiveness in estimating truth, indeterminacy, and falsehood), and robustness to noise (how noise or occlusion affects both accuracy and uncertainty estimation).

**Results:** Table 5 presents a comparative analysis of Type-1 and Type-2 neutrosophic models under various experimental conditions in sign language recognition. In the baseline scenario with known signers and no noise, the Type-2 model achieved a slightly higher accuracy (95%) compared to Type-1 (93%) while maintaining low uncertainty estimates for both models. However, in subject-independent validation with unseen signers, Type-2 demonstrated a notable improvement in accuracy (88%) over Type-1 (80%) and maintained a lower accuracy drop due to uncertainty. In noise simulation with blurred hands, Type-1 experienced a more significant accuracy drop (25%) and had lower accuracy (70%) compared to Type-2, which achieved 82% accuracy with a medium-high uncertainty estimate. Similarly, in the occlusion condition, Type-1’s accuracy further decreased to 68%, reflecting a higher drop (30%), whereas Type-2 maintained a stronger performance at 80% accuracy with a medium uncertainty estimate. Overall, the Type-2 model shows superior performance across various metrics, particularly in challenging conditions like subject independence, noise, and occlusion.

**Justification of Results:** The results indicate that the Type-2 neutrosophic model outperforms Type-1 across all conditions due to its superior handling of uncertainty, leading to higher accuracy, especially with unseen signers and noisy data. While the Type-1 model has crisp boundaries that limit its uncertainty expression, Type-2 offers a more nuanced estimation, making it more suitable for subject-independent tasks. Additionally, Type-2 demonstrates greater robustness to noise and occlusion by incorporating uncertainty through upper and lower bounds in its membership functions, whereas Type-1’s lack of flexibility results in a sharper accuracy drop in challenging conditions.

Experiment 2: Effect of Membership Function Designs on Accuracy in Sign Language Recognition

**Aim:** The goal of this experiment is to evaluate the impact of different membership function designs (trapezoidal, triangular, and Gaussian) on the accuracy of a Type-2 Neutrosophic HMM for sign language recognition.

**Procedure:** Configure three models: Model A, a Type-2 Neutrosophic HMM utilizing trapezoidal membership functions for truth, indeterminacy, and falsehood; Model B, using triangular membership functions; and Model C, employing Gaussian membership functions. Following this, divide the dataset into training (80%) and testing (20%) subsets. Train all three models on the training set and assess their performance by evaluating accuracy on the testing set, which will serve as the primary metric for comparison among the models.

**Results:** Table 6 presents the accuracy results of three different Type-2 Neutrosophic HMMs using various membership function designs for sign language recognition. Model A, which utilizes trapezoidal functions, achieved the highest accuracy at 92%, indicating its effectiveness in capturing and representing uncertainty in sign recognition tasks. In contrast, Model B, based on triangular functions, yielded a lower accuracy of 85%, suggesting that the crisp boundaries of triangular membership functions may limit its capability to handle variability effectively. Model C, employing Gaussian functions, demonstrated a solid performance with an accuracy of 90%, showcasing its balance between flexibility and precision. Overall, these results highlight the significant impact of membership function design on model performance in handling uncertainty in sign language recognition.

**Justification of Results:** The results demonstrate that the choice of membership function significantly affects the accuracy of the Type-2 Neutrosophic HMM in sign language recognition. Model A, using trapezoidal functions, achieved the highest accuracy (92%) due to its flexibility in representing uncertainty through broader and smoother transitions between membership levels. This allows it to handle variability more effectively. Model B, with triangular functions, produced the lowest accuracy (85%) because the sharp boundaries in triangular membership functions are less effective at capturing the uncertainty and indeterminacy present in sign language data. Model C, utilizing Gaussian functions, achieved a balanced accuracy of 90%, benefiting from its smooth, bell-shaped curves that offer a compromise between sharp transitions and flexibility, making it well-suited for handling moderate levels of uncertainty [7].

Experiment 3: Effect of different FOU values on the accuracy of sign language recognition

**Aim:** The goal of this experiment is to evaluate how different FOU sizes (representing different levels of uncertainty) affect the accuracy of sign language recognition.

**Procedure:** To test the impact of different FOU sizes, you can define small, medium, and large FOU values based on the relative width of the trapezoidal membership functions for the three components (truth, indeterminacy, and falsehood). A small FOU represents tight membership functions with a narrow uncertainty range, offering limited flexibility. A medium FOU uses moderately wide membership functions, balancing uncertainty representation and decision-making boundaries. In contrast, a large FOU employs wide membership functions, allowing for a higher range of uncertainty, which may result in more variability but looser decision boundaries. Keep all other model parameters constant (e.g., learning rate, batch size, number of hidden states in the HMM).

**Results:** The results in Table 7 indicate that the medium FOU size (15%) yields the highest performance across all metrics, with an accuracy of 92.5%, precision of 90.3%, recall of 91.1%, and an F1 score of 90.7%. This suggests that a moderate level of uncertainty allows the model to effectively balance flexibility and decision boundary precision, leading to the best recognition performance. In contrast, the small FOU (5%) provides tighter boundaries, resulting in slightly lower accuracy (87.0%) and F1 score (85.0%), as it may underrepresent variability in the data. The large FOU (30%) exhibits the lowest performance, with an accuracy of 82.3% and a corresponding drop in precision, recall, and F1 score, likely due to overly broad uncertainty representation, leading to less defined decision-making.

**Justification of Results:** The results in the table can be justified by the relationship between the FOU size and the model’s ability to handle uncertainty in sign language recognition. A small FOU (5%) creates tight decision boundaries, resulting in decent accuracy (87.0%) but slightly lower precision and recall, as it may struggle to account for variability in the data. The medium FOU (15%) offers the best performance across all metrics, with the highest accuracy (92.5%) and F1 score (90.7%), as it strikes an ideal balance between accommodating natural variability and maintaining precise decision boundaries. In contrast, a large FOU (30%) allows too much uncertainty, which leads to a decline in accuracy (82.3%) and precision (80.0%), as the broader uncertainty range causes the model to overgeneralize, making it less effective at distinguishing between different signs. This demonstrates that a moderate FOU size is optimal for achieving high performance in sign language recognition.

Experiment 4: Justifying the experiment on temporal features for sign language recognition

**Aim:** The goal of this experiment is to verify how different approaches to sign feature extraction—specifically a Kinematic Model and appearance-based low-level features—impact the accuracy of sign language classification.

**Procedure:** In this experiment, three models are trained to evaluate different feature extraction approaches for sign language recognition. The first model, a Kinematic Model, extracts temporal features, like velocity, acceleration, and joint angles of the hands, arms, or fingers, and uses these dynamics alone to classify signs. The second model, an Appearance-Based Model, focuses on low-level visual features such as pixel values, hand contours, shape, and orientation, predicting signs solely from these static features without considering movement. The third is a Combined Model, which integrates both kinematic features (movement dynamics) and appearance-based features (visual characteristics like hand position) to create a more comprehensive classification system. All models are evaluated using accuracy, precision, recall, and F1 scores to compare how well they capture the dynamics and structure of sign gestures.

**Results:** The results in Table 8 show that the Combined Model, which incorporates both kinematic (temporal) and appearance-based features, significantly outperforms the other models, achieving the highest accuracy (93.2%), precision (91.8%), recall (92.5%), and F1 score (92.1%). This indicates that combining dynamic movement information (such as velocity and acceleration) with visual characteristics (like hand shape and position) provides a more comprehensive understanding of sign language gestures. The Kinematic Model, which relies solely on temporal features, performs well with an accuracy of 88.0%, as it effectively captures gesture dynamics but misses some critical visual details. The Appearance-Based Model, focused only on static visual features, achieves the lowest accuracy (85.5%), showing that static features alone are insufficient for recognizing dynamic gestures. Overall, the combined approach proves most effective for sign language recognition by leveraging both motion and visual aspects.

**Justification of Results:** The results in the table can be justified by the different types of information captured by each model. The Kinematic Model performs well with an accuracy of 88.0% because it captures dynamic features like velocity and acceleration, which are crucial for understanding the movement in sign language gestures. However, it misses important static visual details, such as hand shapes, which limits its overall precision and recall. The Appearance-Based Model achieves the lowest performance (85.5% accuracy) because it only considers static features like hand contour and position, which are insufficient for recognizing gestures that involve complex motion. The Combined Model outperforms both with 93.2% accuracy by integrating both kinematic and appearance-based features, providing a more holistic representation of the gestures. This combination allows the model to better understand both the dynamic motion and visual characteristics of signs, leading to the highest precision, recall, and F1 score. This suggests that a multi-modal approach is crucial for optimal sign language recognition.

### 4.1. System Validation Using Statistical Tests

In the suggested method for subject-independent sign language recognition, it is crucial to validate the statistical significance of the results. This ensures that the observed performance improvements are not due to random chance but are statistically meaningful. To assess the model’s performance, based on the data listed in Table 4, we can perform a statistical analysis that includes One-Way ANOVA to determine significant differences in accuracy across experimental conditions, model types, and confidence intervals (CIs) to quantify uncertainty and provide a range for accuracy values.

- One-Way ANOVA: We utilized One-Way ANOVA to compare the accuracy across the four conditions (Baseline, Subject-Independent, Noise Simulation, and Occlusion) for Type-1 and Type-2 models separately. This will help us determine whether the model performance is significantly affected by the experimental condition or the model type.
  - **Null Hypothesis H_0_.** *There is no significant difference in the accuracy across the different experimental conditions for each model type*.
  - **Alternative Hypothesis H_1_.** *At least one experimental condition has a significantly different accuracy*.

The results are listed in Table 9. Since the *p*-values for both Type-1 and Type-2 are below 0.05, we reject the null hypothesis for both model types, suggesting that the accuracy varies significantly across the different conditions.

- Confidence Intervals (CIs): For each condition and model type, we calculated 95% CIs around the accuracy to estimate the range of likely values for the true population accuracy.

The confidence intervals shown in Table 10 give us a sense of the range of likely accuracy values. For example, the accuracy for Type-1 in the Noise Simulation condition ranges from 65% to 75%, which indicates a considerable uncertainty compared to Type-2, which ranges from 78% to 85%.

### 4.2. Real-World Implementation and Testing Example

The real-world implementation of the proposed model for SISLR holds significant potential to revolutionize accessibility and inclusivity for individuals relying on sign language for communication. By addressing uncertainty in recognition and enhancing subject independence, the model can be seamlessly integrated into various real-life applications to improve interactions, learning, and accessibility for the hearing-impaired community. Practical examples of deployment in assistive devices, educational tools, and customer service automation can illustrate its transformative impact:

- Assistive Devices for the Hearing Impaired: Wearable or portable devices, such as smart glasses or mobile applications, can leverage the suggested model to interpret sign language in real time. These devices can translate gestures into text or speech, allowing users to engage in seamless communication with non-signers. For instance, a mobile app equipped with the model could capture gestures via the phone’s camera and display text translations instantly. Similarly, smart glasses can provide augmented reality overlays that convert signs into subtitles for real-time conversations, making everyday interactions more inclusive.
- Educational Tools: The model can be deployed in schools or training centers to facilitate sign language learning for both students and educators. For example, an interactive learning application powered by the proposed model could recognize students’ gestures and provide real-time feedback on accuracy and fluency. This would help students refine their skills and boost their confidence. Teachers, especially those unfamiliar with sign language, could use such tools to better communicate with their students through dynamic translation and gesture recognition systems. These tools can also assist in bridging gaps in inclusive education.
- Customer Service Automation: The suggested model can be integrated into service kiosks or virtual assistants to enhance accessibility in both public and private sectors. For instance, at airports, shopping malls, or hospitals, interactive kiosks equipped with gesture recognition capabilities can help hearing-impaired users access services independently. Virtual assistants, enhanced with sign language recognition, can be deployed online or in physical locations to ensure that communication barriers are eliminated, providing a seamless user experience for customers requiring sign language support.

To validate the proposed model, real-world testing should include diverse scenarios. Public accessibility trials in government offices, banks, and airports can assess metrics like accuracy, response time, and user satisfaction through interactive service kiosks. In classroom settings, the system can be tested for its ability to enhance communication between teachers and students with varied signing styles. A real-time translation app prototype providing sign-to-text or sign-to-speech functionality can be evaluated in everyday situations, such as ordering food or asking for directions. Field studies with assistive devices, like smart glasses or wearables, can measure accuracy, usability, and comfort in dynamic environments, including workplaces and social gatherings. The model’s adaptability can be gauged through cross-language testing with multiple sign languages, while multi-user interaction scenarios can assess robustness against overlapping gestures or background interference.

## 5. Conclusions

The proposed Type-2 Neutrosophic HMM for subject-independent sign language recognition (SISLR) represents a significant advancement in addressing the challenges posed by variability and imprecision in sign language gestures. By leveraging the Footprint of Uncertainty (FOU) inherent in Type-2 neutrosophic sets, the model effectively captures and manages the uncertainty in truth, falsity, and indeterminacy, resulting in a more robust and accurate recognition system. The experimental results demonstrate that the Type-2 Neutrosophic HMM consistently outperforms the Type-1 Neutrosophic HMM, achieving an impressive 10% increase in average accuracy across diverse test scenarios.

The enhanced accuracy, superior handling of complex uncertainties, and improved robustness of the proposed system make it particularly suited for applications in real-time sign language translation systems, accessibility tools for the deaf and hard-of-hearing community, and automated sign language transcription for education and workplace integration. Additionally, its ability to handle diverse signing conditions positions it as a promising tool for international sign language recognition systems, where variability in regional and cultural gestures adds complexity.

Future work will focus on enhancing the system’s practicality and scalability by reducing computational complexity through algorithm optimization and leveraging hardware accelerators like GPUs, ensuring efficient processing for deployment on low-power devices such as mobile phones and wearables. Real-time performance will be improved by streamlining feature extraction and inference with lightweight architectures or pruning techniques, enabling low-latency processing for live translation and interactive aids. Scalability will be achieved through advanced training strategies like incremental and transfer learning to accommodate larger datasets and new signs without retraining, alongside developing a modular framework for multilingual and region-specific integration. Additionally, integrating the system with cloud-based platforms and distributed frameworks will support large-scale deployments, paving the way for applications in education, workplace accessibility, and assistive technologies for the deaf and hard-of-hearing community.

## Figures and Tables

**Table 1:** A comparison between Type-1 Neutrosophic Logic and Uncertainty-Aware Deep Neural Networks.

Aspect	Type-1 Neutrosophic Logic (T1NL)	Uncertainty-Aware Deep Neural Networks (UADNNs)
Handling Uncertainty	Models truth, indeterminacy, and falsity, handling ambiguity effectively.	Quantifies uncertainty through probabilistic methods and confidence scores.
Complexity	Moderate complexity, involving degrees of truth, indeterminacy, and falsity.	High complexity due to advanced neural network architectures and uncertainty models.
Scalability	Can be less scalable to high-dimensional or complex datasets.	Scalable but may face challenges with very large datasets or high-dimensional data.
Computational Overhead	Generally lower computational overhead compared to deep learning models.	High computational cost due to deep learning model training and inference.
Real-time Performance	May struggle with real-time performance due to computational requirements.	Potentially suitable for real-time applications, but depends on model complexity.
Interpretability	Provides clear interpretability with uncertainty components.	Interpretation can be challenging, often requiring understanding of probabilistic outputs.
Accuracy with High Variability	May struggle with high variability in sign language data.	Generally performs well with high variability due to deep learning capabilities.
Flexibility	Limited adaptability to different scenarios without extensive adjustments.	Highly adaptable to various scenarios with advanced architectures and techniques.
Integration with Existing Systems	Can be integrated with traditional systems but may require specialized adaptation.	Easily integrable with existing deep learning frameworks and tools.
Suitability for Noisy Data	Effective in managing noisy data through uncertainty representation.	Effective in noisy environments with probabilistic models but requires robust training.

**Table 2:** A comparison between Type-2 Neutrosophic Logic for SLR with other uncertainty-handling techniques.

Feature	Type-2 Neutrosophic HMM	Traditional Fuzzy Logic	Probability-Based Approaches	Type-1 Neutrosophic Systems
Uncertainty Representation	Three dimensions: truth, falsity, and indeterminacy, using intervals (FOU) for second-order uncertainty	Single membership degrees, no higher-order uncertainty	Uses probability distributions or point estimates, no multi-dimensional uncertainty	Truth, falsity, and indeterminacy but no FOU, single-level uncertainty
Handling Ambiguity	Captures a range for each uncertainty level, managing ambiguous gestures with interval-based FOU	Limited flexibility in ambiguity; adjustments needed for uncertain cases	Probability-based confidence scores, struggles with highly ambiguous cases	Represents truth, falsity, and indeterminacy, but less adaptable than Type-2
Adaptability to Variability	Adapts to diverse gestures, signing styles, lighting, occlusions, and background changes	Recalibration often required for diverse input	Sensitive to data quality; requires substantial labeled data for variability	Can handle variability but lacks interval adaptability seen in Type-2
Dependence on Data Volume	Lower dependency; interval-based uncertainty handling reduces need for extensive labeled data	Moderate dependency, especially for complex systems	High dependency on large datasets for reliable probability estimates	Moderate; less data-dependent than probability-based but more than Type-2
Resistance to Model Drift	High stability against model drift; adapts over time without frequent retraining through FOU	Low resistance to drift, requiring periodic recalibration	Susceptible to drift; requires frequent updates as patterns change	Moderate resistance to drift; needs tuning but more stable than probability-based
Real-Time Scalability	Computationally efficient for real-time applications due to interval-based FOU, reducing recalibration need	Computationally demanding in complex environments	High computational load with larger datasets and model complexity	Moderate scalability, less efficient than Type-2, often requires tuning
Complexity of Uncertainty Handling	Advanced, captures first- and second-order uncertainties with interval representation in FOU	Single-layer uncertainty representation	Simple probability estimates; lacks nuanced multi-level uncertainty handling	Handles multiple dimensions but lacks detailed interval flexibility in Type-2
Applicability to SLR	Suitable for high-variability cases in SLR, e.g., different styles, lighting, occlusions	Limited applicability to diverse environments in SLR	Useful for specific, well-defined patterns but struggles with SLR variability	Useful in SLR, but may need additional calibration for environmental changes

**Table 3:** Analysis of methods discussed for SLR: strengths and limitations.

Approach	Methodology	Pros	Cons
Gaussian Processes (GPs)	Non-parametric probabilistic framework for quantifying uncertainty in SLR.	- Effective in detecting ambiguous or novel gestures. - Adaptable to various sign data distributions. - Provides inherent uncertainty estimates.	- Scalability challenges with large datasets due to quadratic covariance matrix. - Computationally intensive during training and inference. - Struggles with high-dimensional data or complex sequences.
Bayesian Neural Networks (BNNs)	Neural networks that handle both epistemic and Aleatoric uncertainty, quantifying model and data uncertainty.	- Robust in handling uncertainty. - Provides confidence intervals for predictions. - Reduces overfitting in small datasets.	- High computational costs due to complex inference methods like Monte Carlo sampling. - Scalability challenges. - May have lower raw accuracy compared to deterministic models.
Ensemble Methods (Bootstrap Aggregating)	Combines multiple models to estimate uncertainty and reduce overfitting.	- Enhances uncertainty estimation. - Reduces overfitting. - Effective with ambiguous inputs.	- High computational cost due to training multiple models. - Resource demands can affect real-time performance. - Requires careful tuning, with marginal performance gains in some cases.
Deep Evidential Regression	Direct uncertainty prediction with deep learning, used for real-time SLR.	- Efficient uncertainty quantification in a single pass. - Suitable for real-time applications. - Improves reliability in noisy situations.	- Complex to interpret and tune. - Requires expertise in evidential theory. - Limited performance information for large-scale or diverse datasets. - Prone to overfitting without regularization.
Type-1 Neutrosophic Logic Models	Models uncertainty using truth, indeterminacy, and falsity to handle ambiguous or noisy signs.	- Handles uncertainty effectively. - Enhances decision-making in unclear situations. - Suitable for occlusion, lighting variations, and signer differences.	- Limited ability to handle higher-order uncertainties. - Computational overhead may hinder real-time performance. - May not generalize well with highly variable data.
Bidirectional Translation Framework (Ref. [26])	Converts Arabic sign images into text and vice versa, using CNNs and fuzzy string matching for translation.	- Bridges communication gap between deaf and hearing individuals. - Uses pre-trained CNNs, enhancing efficiency. - Novel fuzzy string matching improves accuracy.	- Relies heavily on the quality of CNNs. - Limited generalization to other languages. - Performance depends on dataset quality.
Image-Based Method (Ref. [27])	Multi-phase method with image processing, feature extraction, classification, and NLP for SLR.	- Effective image processing techniques. - High accuracy in the classification of signs. - Uses NLP for word display.	- Depends on the quality of image processing and skin detection. - Limited to 20 videos for testing. - Framework implemented in MATLAB, which may limit flexibility for real-time applications.

**Table 4:** Three-dimensional kinematic data for samples of ASL alphabet hand signs.

Hand Sign	Position (P)	Orientation (O)	Key Joint 1 (Wrist)	Key Joint 2 (Knuckle)	Key Joint 3 (Middle Finger)	Key Joint 4 (Fingertip)	Key Joint 5 (Thumb Base)
A	(10, 15, 20)	(30°, 45°)	(10, 15, 20)	(11, 16, 19)	(13, 18, 18)	(14, 20, 17)	(9, 16, 21)
B	(12, 16, 18)	(20°, 60°)	(12, 16, 18)	(13, 18, 17)	(14, 19, 16)	(15, 20, 15)	(11, 17, 19)
C	(11, 14, 21)	(25°, 40°)	(11, 14, 21)	(13, 15, 22)	(14, 17, 23)	(15, 19, 22)	(10, 15, 20)
D	(9, 14, 19)	(35°, 55°)	(9, 14, 19)	(10, 16, 20)	(12, 17, 18)	(13, 18, 17)	(8, 16, 20)
E	(10, 13, 22)	(30°, 50°)	(10, 13, 22)	(11, 15, 23)	(13, 16, 24)	(14, 17, 25)	(9, 12, 21)
F	(11, 15, 19)	(40°, 45°)	(11, 15, 19)	(12, 16, 18)	(13, 17, 17)	(14, 18, 16)	(10, 14, 20)
G	(13, 17, 20)	(50°, 30°)	(13, 17, 20)	(14, 19, 22)	(15, 20, 23)	(16, 21, 22)	(12, 18, 19)
H	(12, 16, 18)	(45°, 40°)	(12, 16, 18)	(13, 18, 20)	(14, 19, 21)	(15, 20, 22)	(11, 17, 19)
I	(10, 14, 19)	(35°, 50°)	(10, 14, 19)	(11, 15, 21)	(12, 16, 22)	(13, 17, 23)	(9, 13, 20)
K	(11, 15, 18)	(30°, 45°)	(11, 15, 18)	(12, 17, 19)	(13, 18, 20)	(14, 19, 21)	(10, 16, 19)

**Table 5:** Performance comparison of Type-1 and Type-2 neutrosophic models in subject-independent sign language recognition under varying conditions.

Experiment Condition	Model Type	Accuracy (%)	Uncertainty Estimate (High/Medium/Low)	Robustness to Noise (Accuracy Drop %)
Baseline (Known Signers, No Noise)	Type-1	93%	Low	2%
	Type-2	95%	Low	1%
Subject-Independent Validation (Unseen Signers)	Type-1	80%	Medium	15%
	Type-2	88%	Medium	10%
Noise Simulation (Blurred Hands)	Type-1	70%	High	25%
	Type-2	82%	Medium-High	18%
Occlusion (Partial Visibility of Hands)	Type-1	68%	High	30%
	Type-2	80%	Medium	20%

**Table 6:** Accuracy comparison of Type-2 Neutrosophic HMMs with different membership functions using subject-independent dataset.

Model Type	Accuracy (%)
Model A (Trapezoidal Functions)	92%
Model B (Triangular Functions)	85%
Model C (Gaussian Functions)	90%

**Table 7:** Impact of FOU size on sign language recognition model performance using subject-independent dataset.

FOU Size	FOU Range (%)	Accuracy (%)	Precision (%)	Recall (%)	F1 Score (%)
Small FOU	5%	87.0	84.6	85.4	85.0
Medium FOU	15%	92.5	90.3	91.1	90.7
Large FOU	30%	82.3	80.0	80.8	80.4

**Table 8:** Performance comparison of Kinematic, Appearance-Based, and Combined Models in sign language recognition.

Model	Accuracy (%)	Precision (%)	Recall (%)	F1 Score (%)
Kinematic Model (Temporal)	88.0	86.5	87.1	86.8
Appearance-Based Model	85.5	84.2	84.7	84.4
Combined Model (Kinematic + Appearance)	93.2	91.8	92.5	92.1

**Table 9:** ANOVA results for model performance across neutrosophic model types.

Model Type	F-Statistic	*p*-Value
Type-1 neutrosophic model	32.12	0.001
Type-2 neutrosophic model	29.55	0.001

**Table 10:** Confidence intervals for accuracy across experimental conditions and model types.

Experiment Condition	Model Type	95% CI for Accuracy
Baseline (Known Signers, No Noise)	Type-1	(90.5%, 95.5%)
	Type-2	(92.5%, 97.0%)
Subject-Independent Validation	Type-1	(76%, 84%)
	Type-2	(85%, 91%)
Noise Simulation (Blurred Hands)	Type-1	(65%, 75%)
	Type-2	(78%, 85%)
Occlusion (Partial Visibility)	Type-1	(63%, 73%)
	Type-2	(77%, 83%)

## Data Availability

Data are contained within the article.
